# Supplementary figures and images for: Self-organized metabotyping of obese individuals identifies clusters responding differently to bariatric surgery
Source: PLoS One. 2023 Mar 2;18(3):e0279335. doi: 10.1371/journal.pone.0279335 (PMC9980823; doi:10.1371/journal.pone.0279335)

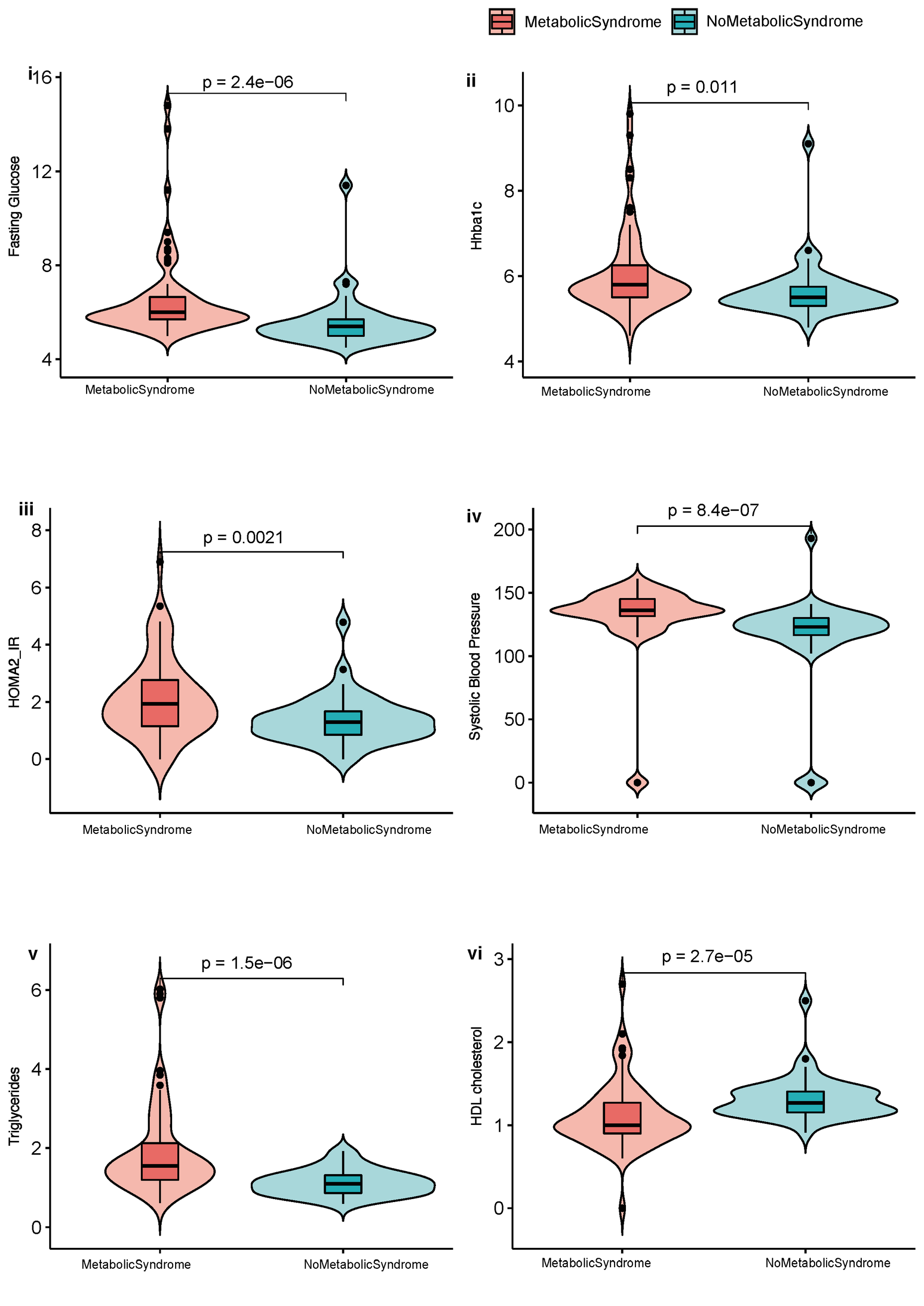

Supplement: S1 Fig — (i) Fasting glucose (ii) Hba1c, (iii) HOMA2_IR, (iv) Systolic Blood Pressure, (v) Triglycerides and (vi) HDL cholesterol statistical significance calculated with Kruskal-Wallis test, symbols indicating significance among metabotypes: ‘*’: P< = 0.05, ‘**’: P< = 0.01, ‘***’: P< = 0.001. (TIF) [file pone.0279335.s001.tif]

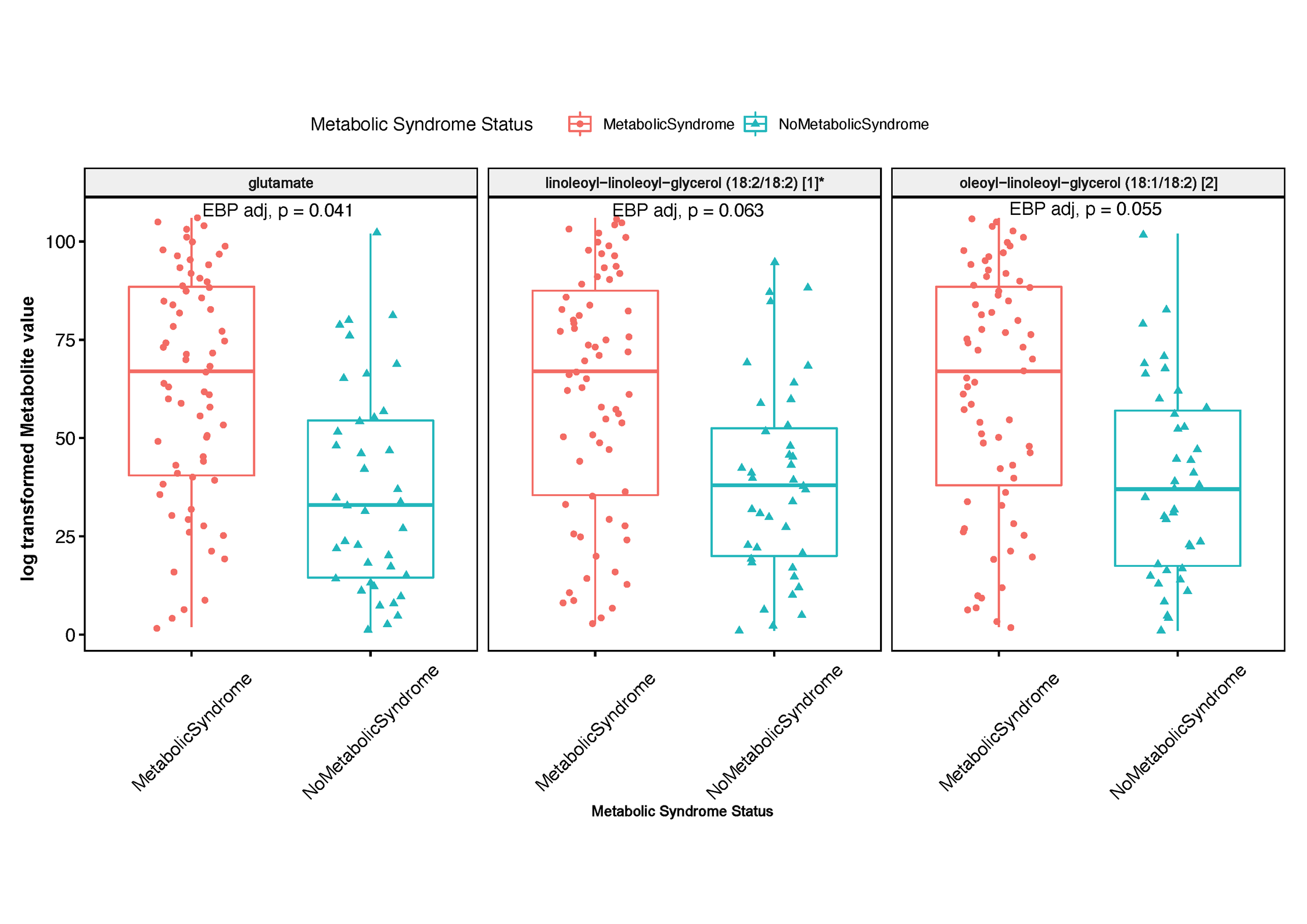

Supplement: S2 Fig — Statistical analysis conducted with HybridMTest and adjusted p value with Estimated Bayesian Probability (P<0.05). (TIF) [file pone.0279335.s002.tif]

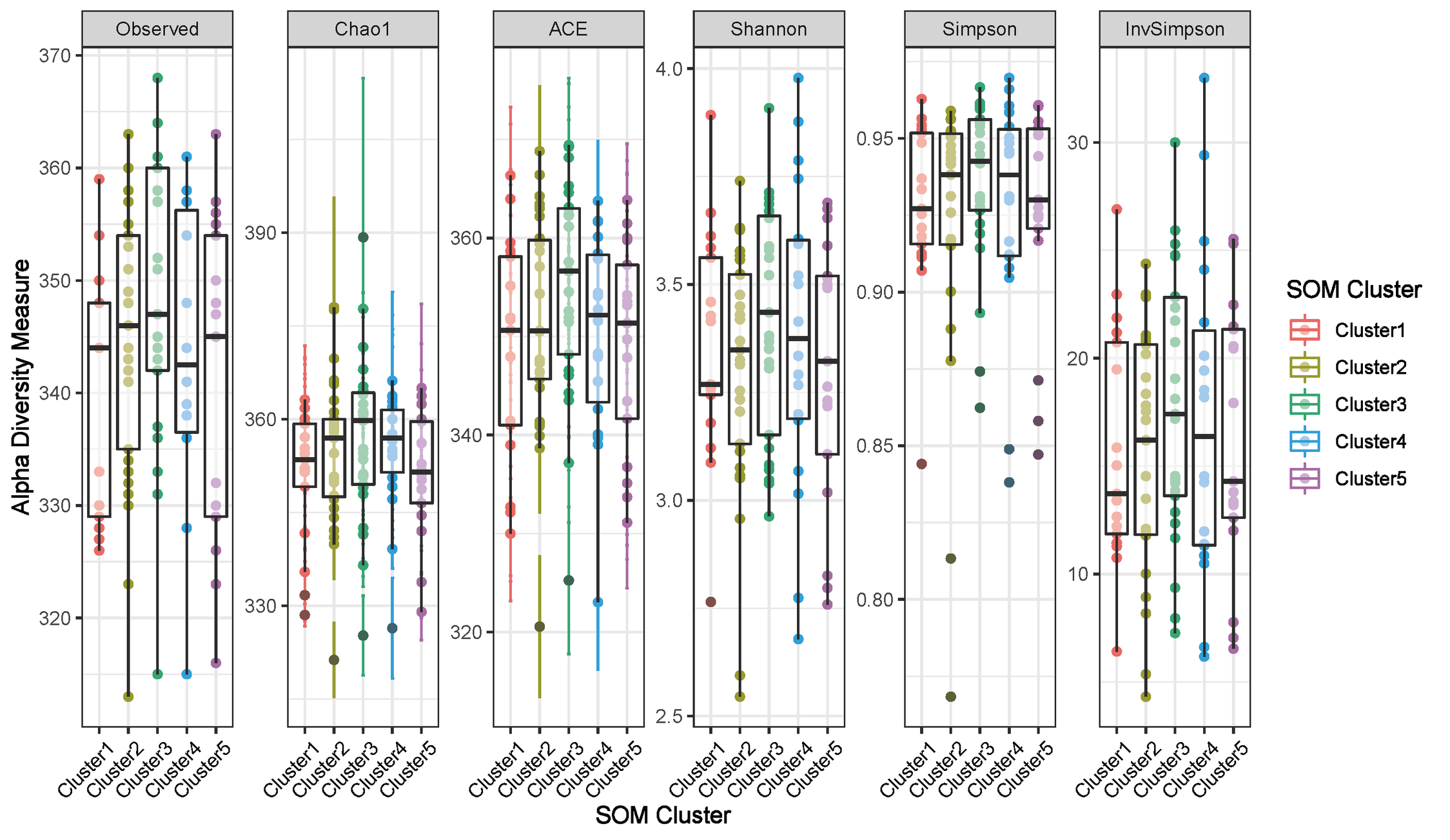

Supplement: S3 Fig — None of the alpha diversity metrics reach statistical significance. (TIF) [file pone.0279335.s003.tif]

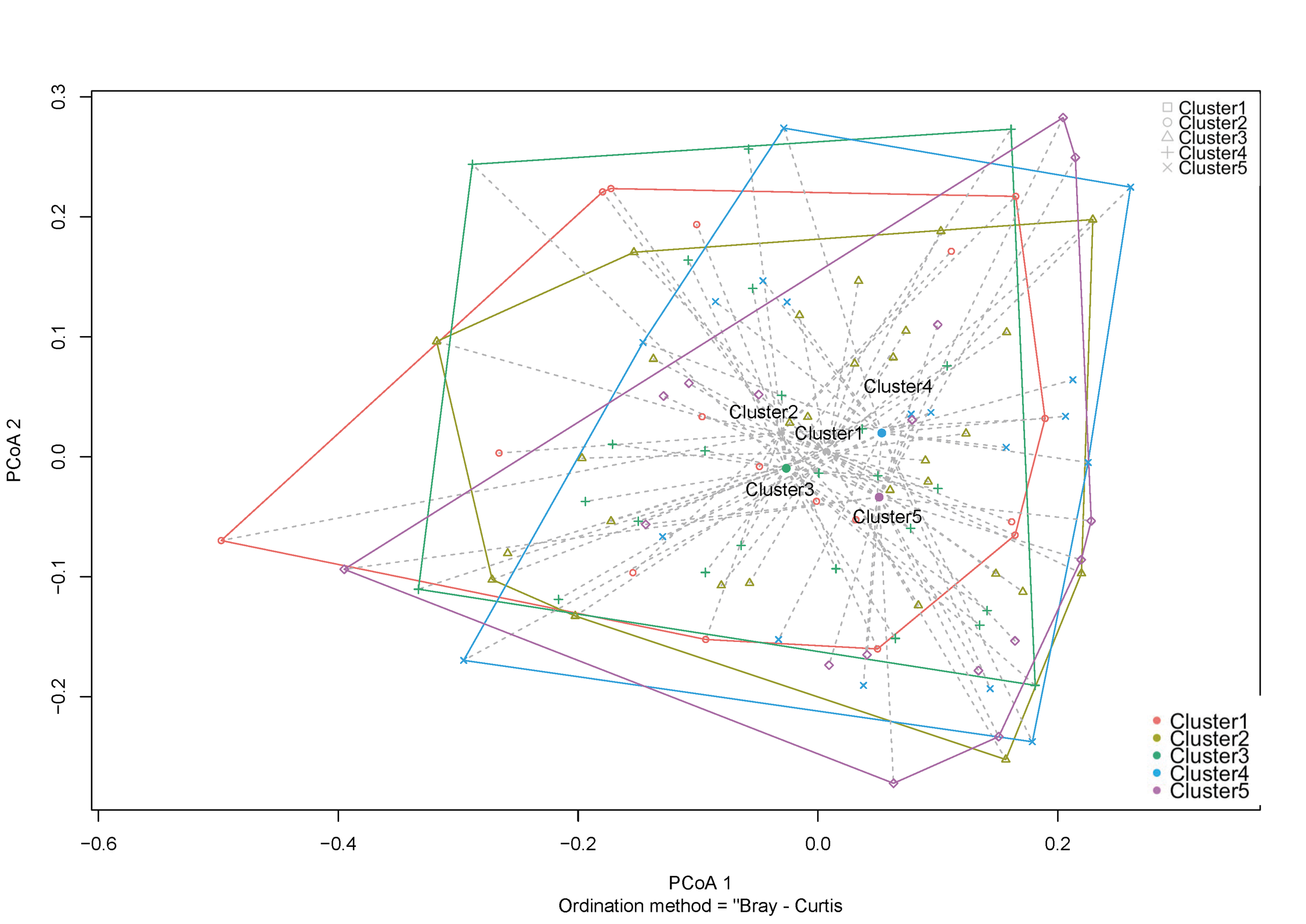

Supplement: S4 Fig — Average Euclidean distances in principal coordinate space between the samples and their respective group centroid are: Cluster1 = 0.3946, Cluster2 = 0.3828, Cluster3 = 0.3777, Cluster4 = 0.3718, and Cluster 5 = 0.3769. The Whittaker diversity index did not reach statistical significance. (TIF) [file pone.0279335.s004.tif]

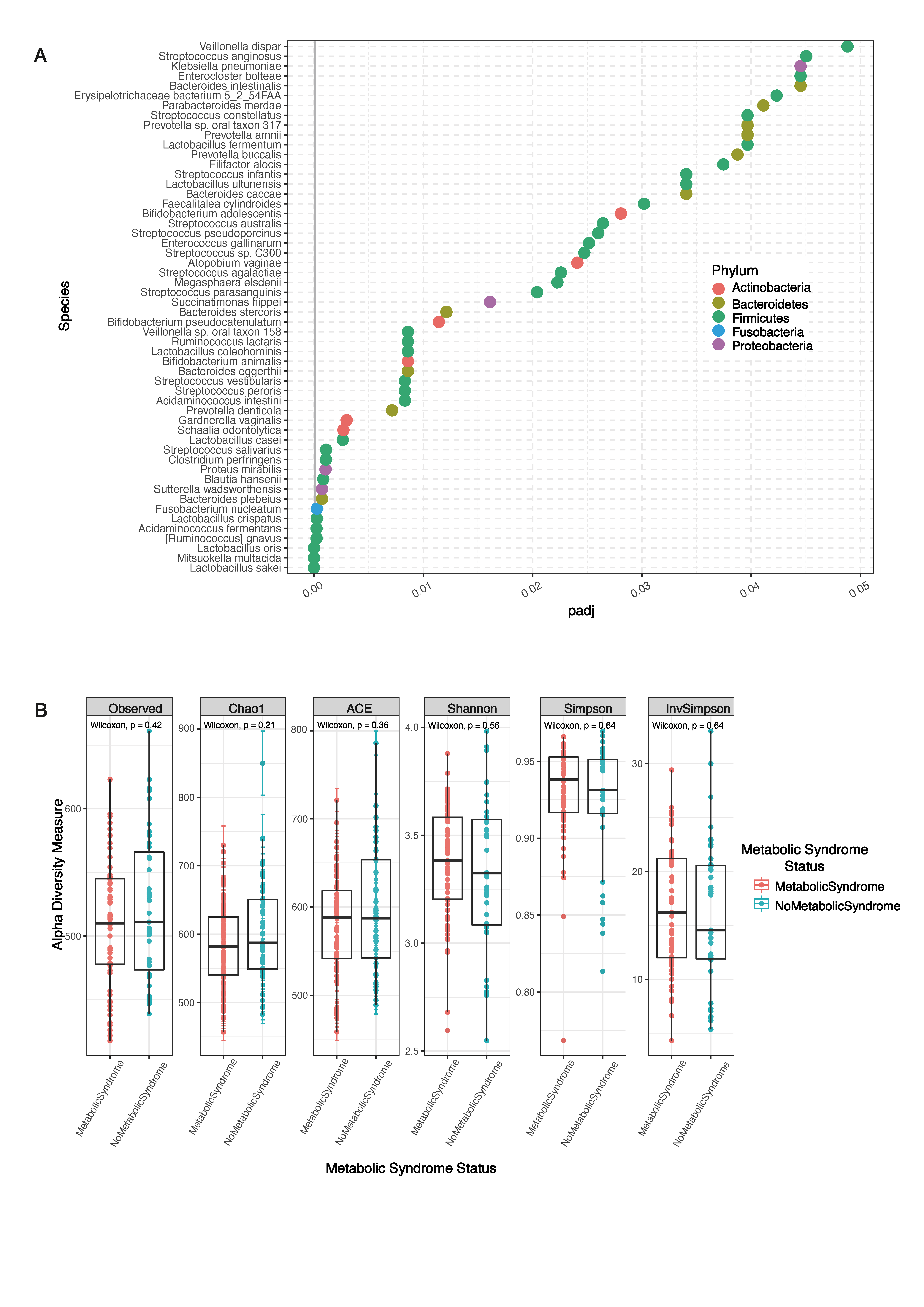

Supplement: S5 Fig — (a) Different measures of microbial species alpha diversity (Observed, Chao1, ACE, Shannon, Simpson, Inverse Simpson) and none of the alpha diversity metrics reach statistical significance. (b) Differentially significant microbial species between BARIA individuals diagnosed with and without Metabolic Syndrome, after statistical analysis with DESeq2 (P<0.05). (TIFF) [file pone.0279335.s005.tiff]

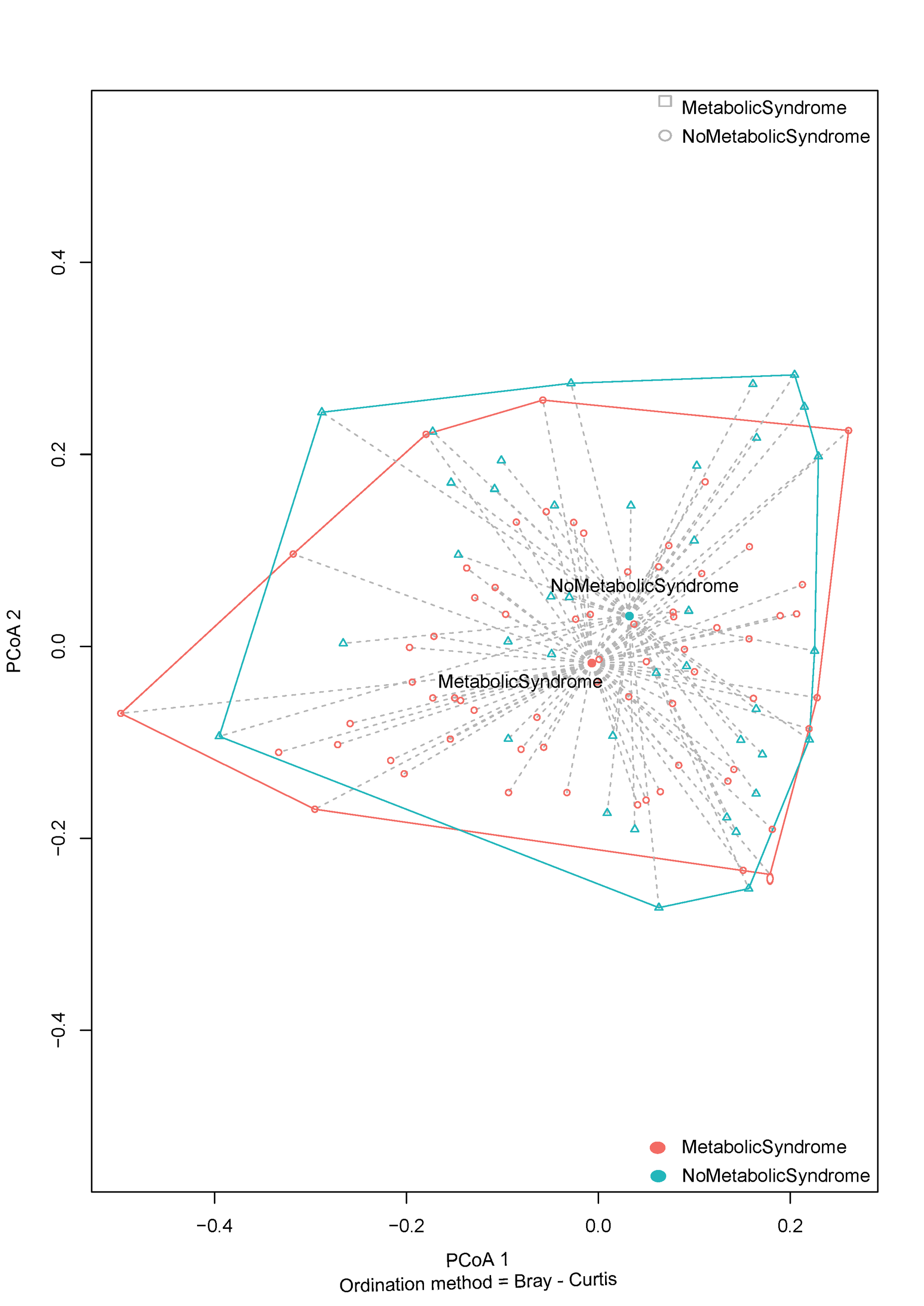

Supplement: S6 Fig — Average Euclidean distances in principal coordinate space between the samples and their respective group centroid are: MetabolicSyndrome = 0.3776 and NoMetabolicSyndrome = 0.3992. The Whittaker diversity index did not reach statistical significance. (TIF) [file pone.0279335.s006.tif]

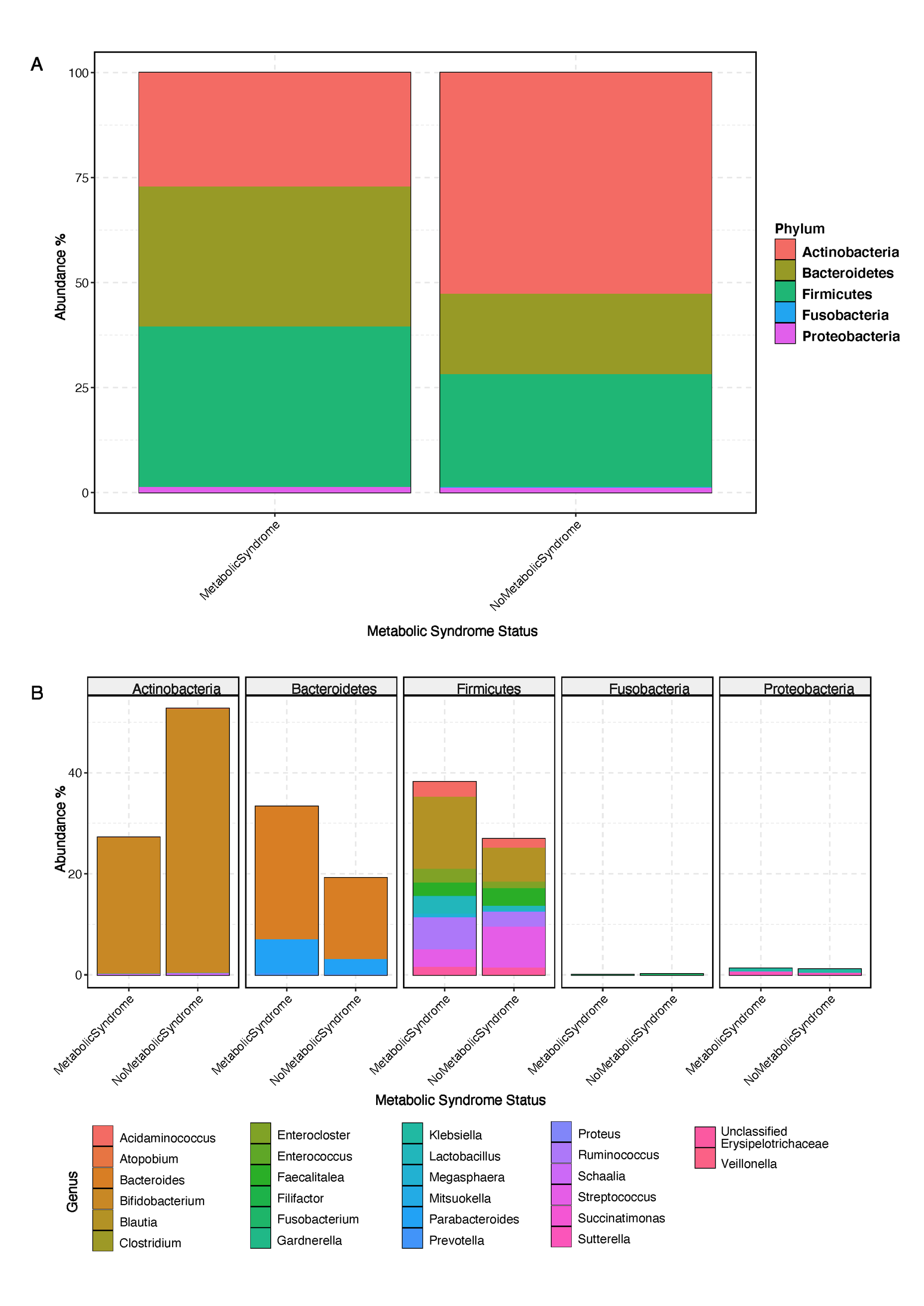

Supplement: S7 Fig — (a) Relative abundance and phyla composition of the differentially significant microbial between BARIA individuals diagnosed with and without Metabolic Syndrome. (b) Distribution of differentially significant microbial species across phyla between BARIA individuals diagnosed with and without Metabolic Syndrome. (TIF) [file pone.0279335.s007.tif]

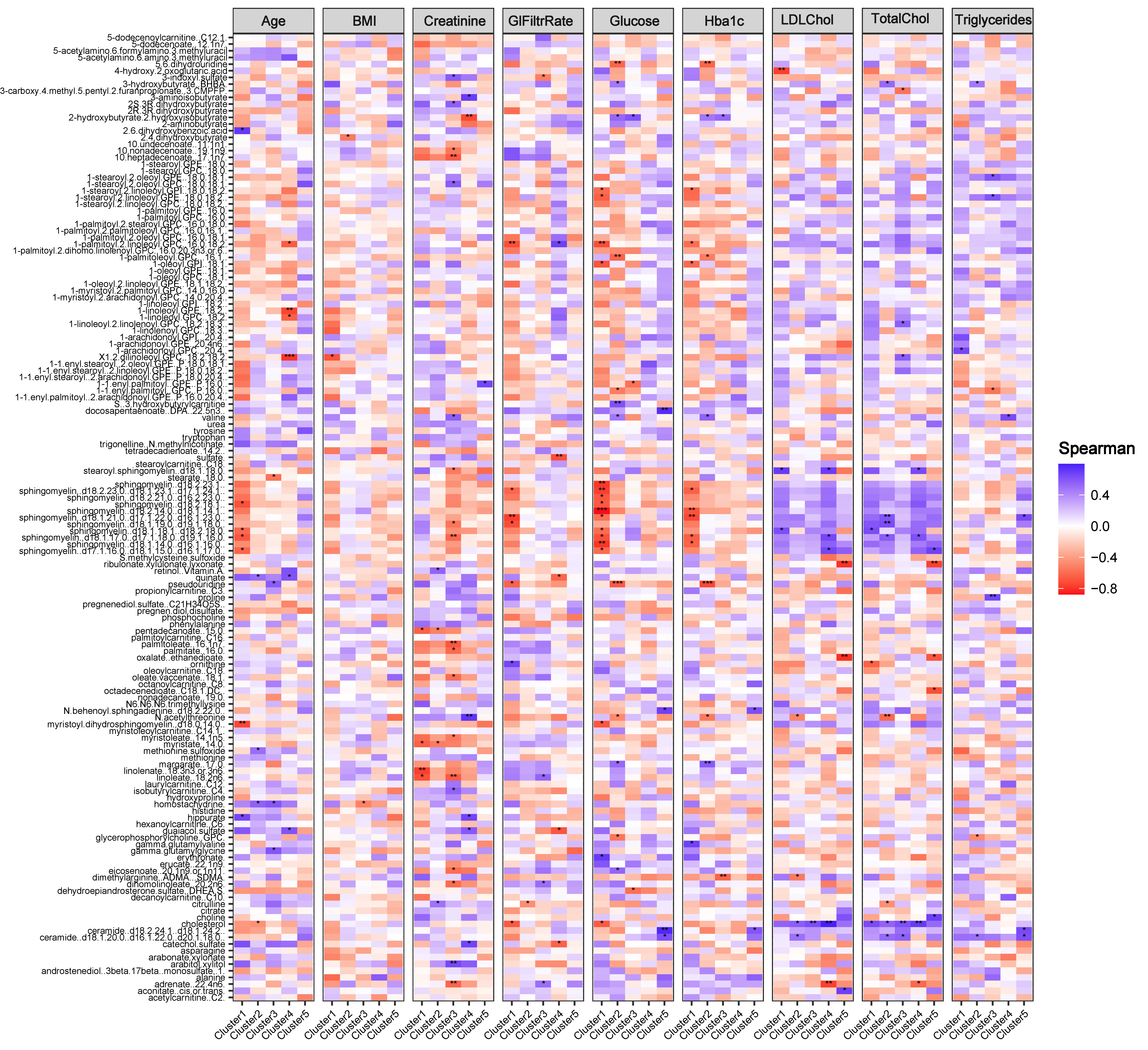

Supplement: S8 Fig — Significance codes are ‘***’ 0.001 ‘**’ 0.01 ‘*’ 0.05 ‘.’ 0.1 ‘ ‘ 1. (TIFF) [file pone.0279335.s008.tiff]

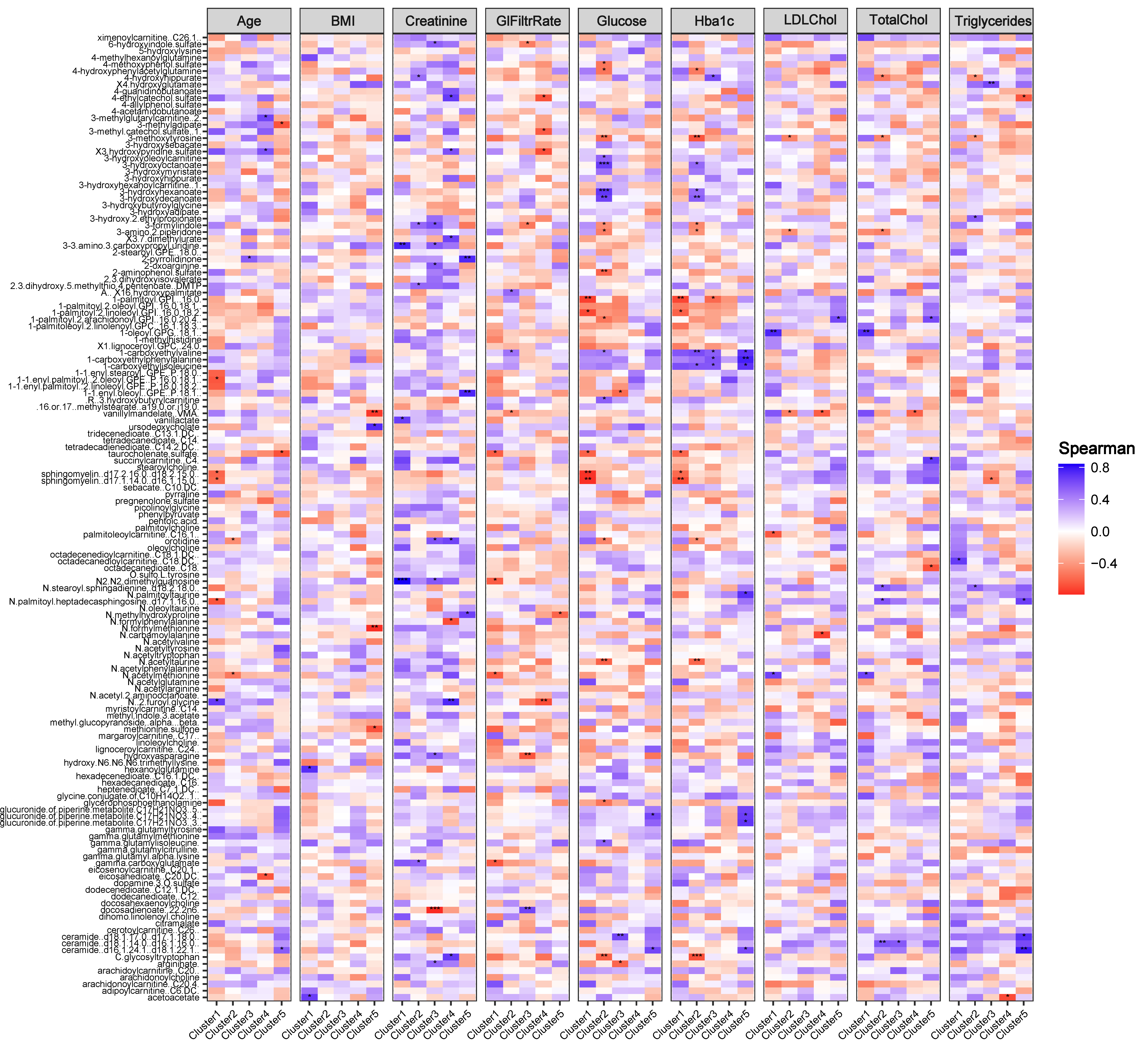

Supplement: S9 Fig — Significance codes are ‘***’ 0.001 ‘**’ 0.01 ‘*’ 0.05 ‘.’ 0.1 ‘ ‘ 1. (TIFF) [file pone.0279335.s009.tiff]

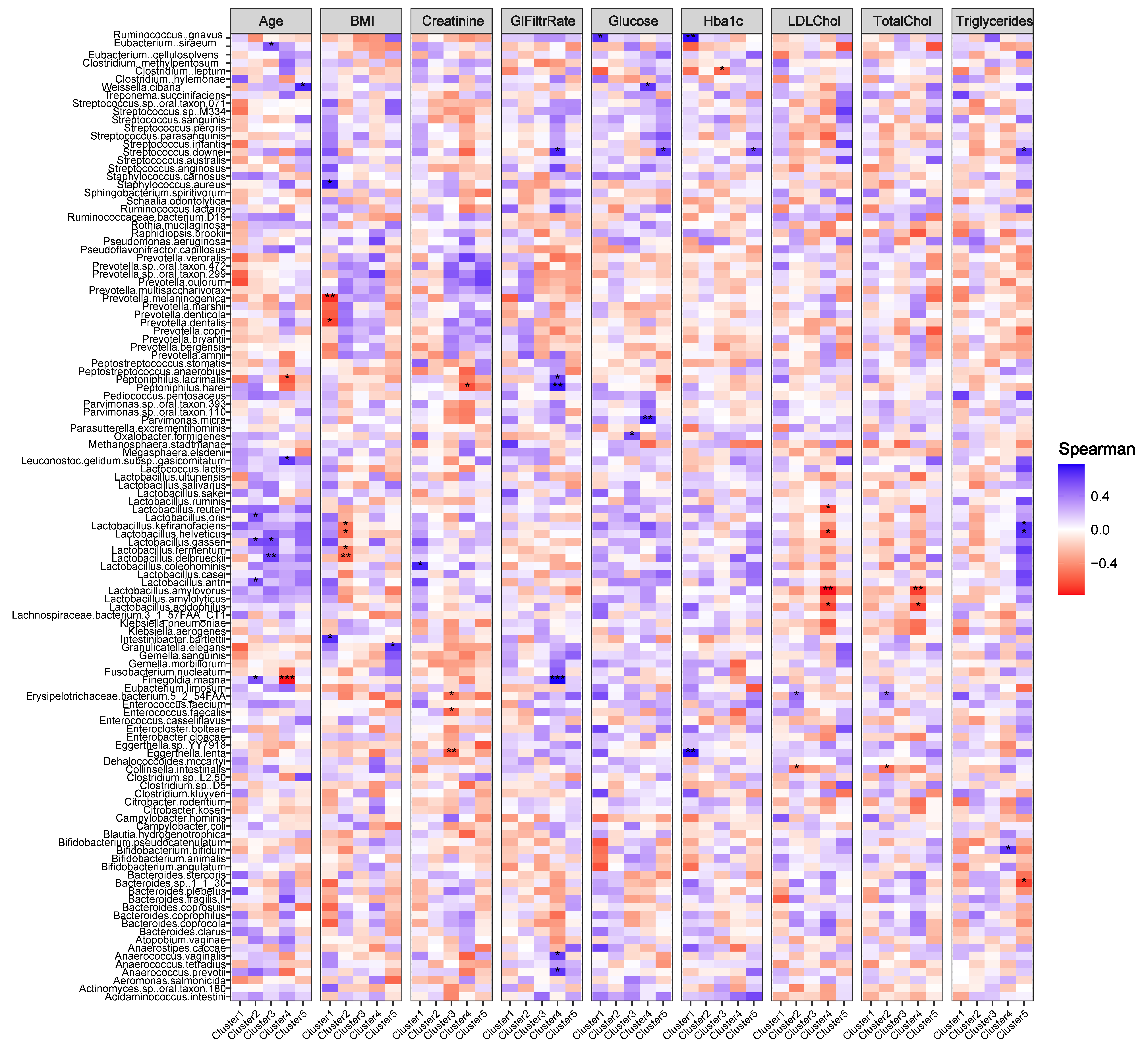

Supplement: S10 Fig — Significance codes are ‘***’ 0.001 ‘**’ 0.01 ‘*’ 0.05 ‘.’ 0.1 ‘ ‘ 1. (TIFF) [file pone.0279335.s010.tiff]

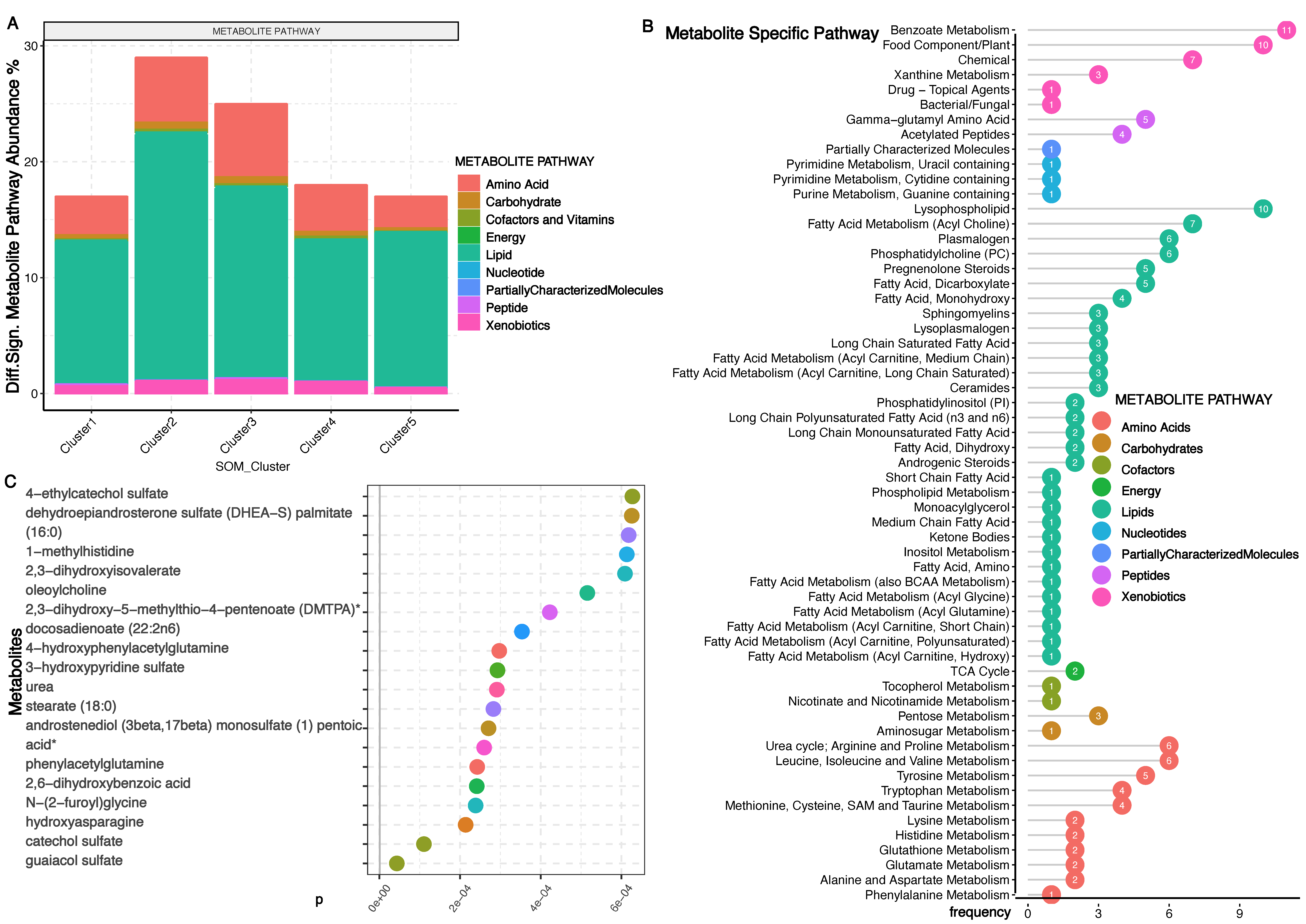

Supplement: S11 Fig — (A) Relative abundance and distribution of differentially significant metabolites among SOM and k-means defined clusters. Clusters two and three are most abundant in lipids (especially lysophospholipids and sphingomyelins) and amino acids (urea, arginine and proline metabolism). (B) Distribution of differentially significant metabolic pathways among SOM and k-means defined clusters, where numbers within each dot indicate how many metabolites of that particular specific pathway were differentially abundant across clusters. (C) Top 20 differentially significant metabolites among the SOM and k-means defined clusters, (P<0.05). (TIFF) [file pone.0279335.s011.tiff]

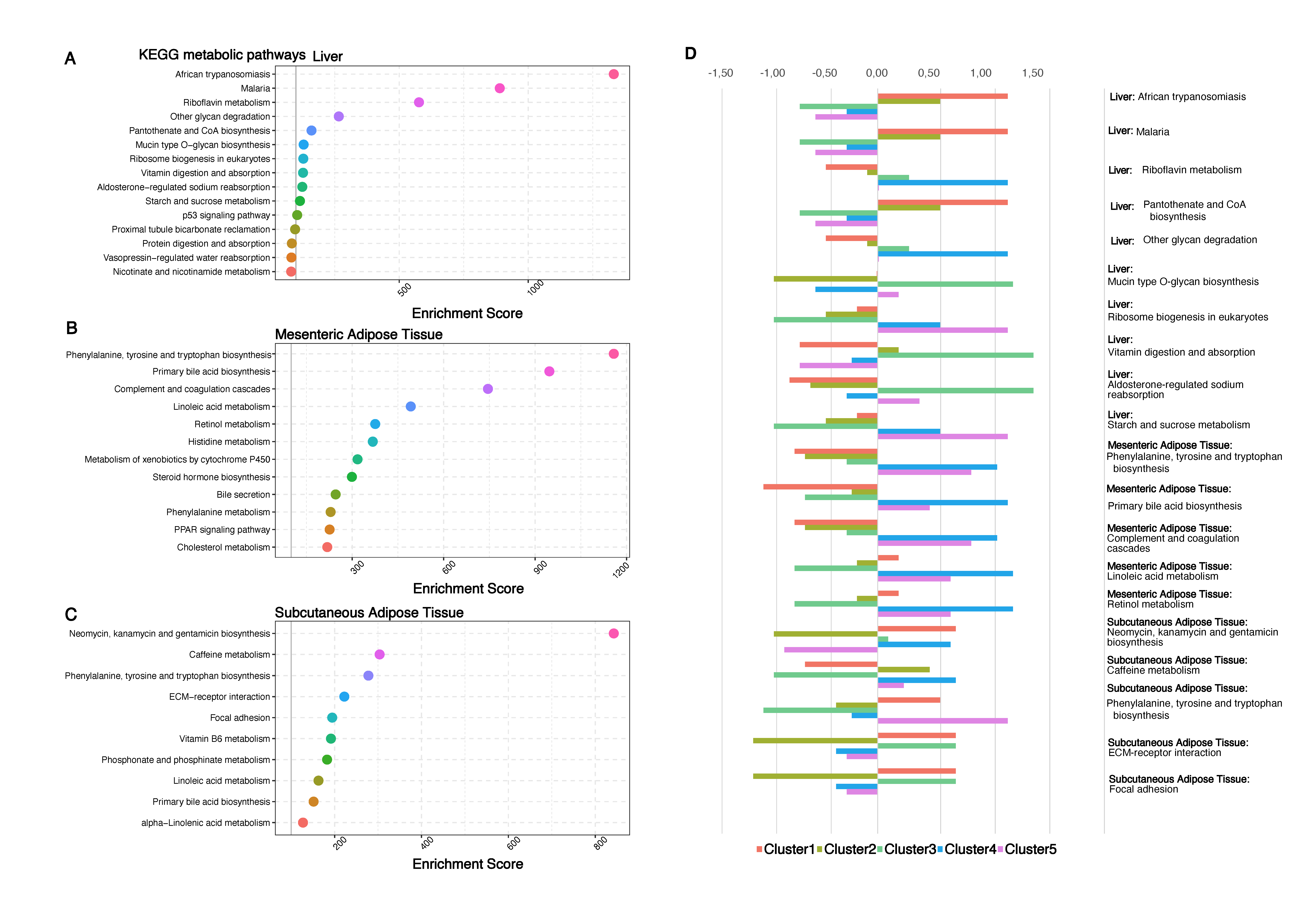

Supplement: S12 Fig — (A) Top 15 differentially enriched KEGG metabolic pathways for hepatic transcriptome among the SOM and k-means defined clusters, ranked based on their scores after differential gene expression analysis (DESeq2, P<0.05) and gene set analysis (GSA with EnrichR). (B) Top 15 differentially enriched KEGG metabolic pathways for mesenteric adipose transcriptome among the SOM and k-means defined clusters, ranked based on their scores after differential gene expression analysis (DESeq2, P<0.05) and gene set analysis (GSA) with EnrichR). (C) Top 10 differentially enriched KEGG metabolic pathways for subcutaneous adipose tissue transcriptome among the SOM and k-means defined clusters, ranked based on their scores after differential gene expression analysis (DESeq2, P<0.05) and gene set analysis (GSA with EnrichR). (D) 20 highest scoring KEGG metabolic pathways according to EnrichR GSA score for liver, mesenteric adipose and subcutaneous adipose tissues. Z score indicates different levels of differentially expressed pathways, for each SOM and k-means defined cluster. (TIFF) [file pone.0279335.s012.tiff]

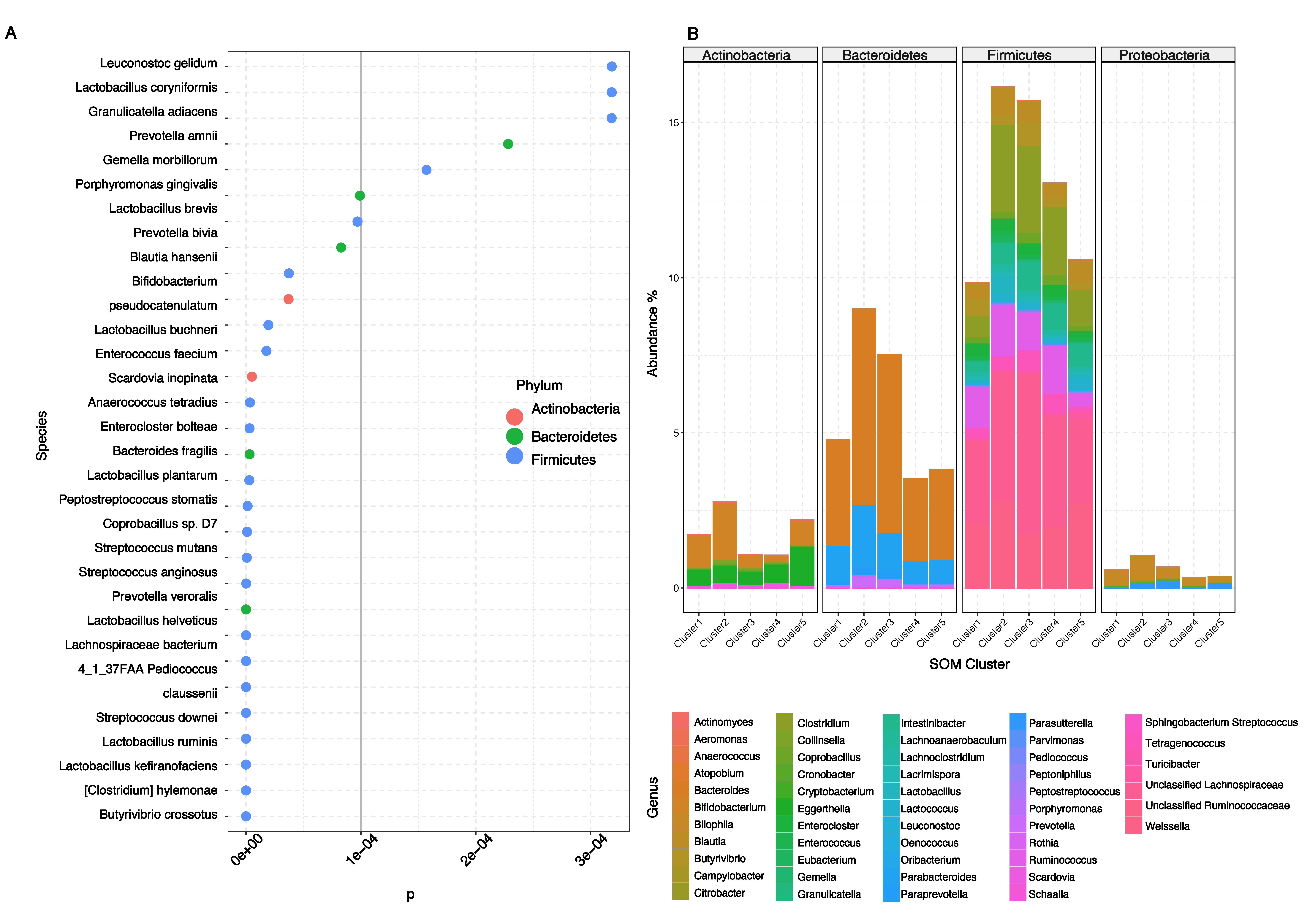

Supplement: S13 Fig — (A) Relative abundance and distribution of differentially significant metabolites among SOM and k-means defined clusters. Clusters two and three are most abundant in lipids (especially lysophospholipids) and amino acids (urea, arginine and proline metabolism). (B) Distribution of differentially significant metabolic pathways among SOM and k-means defined clusters, where numbers within each dot indicate how many metabolites of that particular specific pathway were differentially abundant across clusters. (C) Top 20 differentially significant metabolites among the SOM and k-means defined clusters, (P<0.05). (TIFF) [file pone.0279335.s013.tiff]

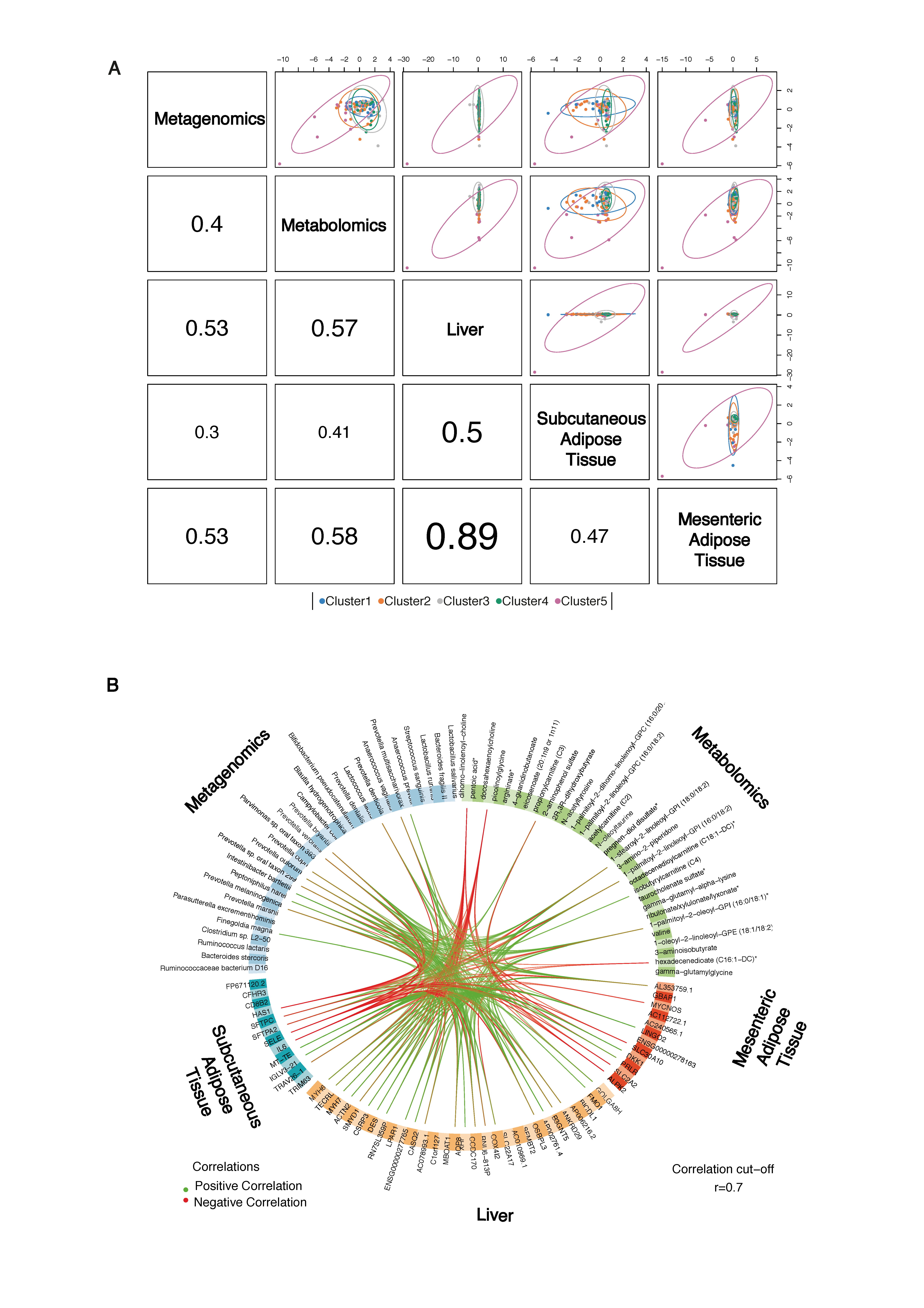

Supplement: S14 Fig — (a) Total correlation matrix for the differentially significant metabolites, genes, microbial species from all the different omic datasets after Sparce Principal Least Squares Regression with mixOmics DIABLO. Highest total dataset correlation was observed for differentially significant genes from the liver RNASeq dataset and mesenteric adipose tissue at r = 0.89, followed by the metabolomics dataset with the liver RNASeq at r = 0.57 and the metagenomics dataset with both liver and mesenteric adipose tissue RNASeq at r = 0.53. (b) Circular correlation plot by DIABLO, for selecting top contributing components from each omics dataset (metabolites, genes, bacterial species). Correlation cut-off was r = 0.7. The chosen elements constituted a highly correlated discriminatory signature for the five metabotypes. This signature involves a series of: i) Prevotella species (P. veroralis, P. copri, P. multisaccharivorax, P. oulorum, P. denticola, P. sp. oral taxon 299, P.bryantii, P. melaninogenica), Intestinibacter bartlettii, Anaerococcus prevotii; ii) lipid metabolites (especially phospatidylcholines); iii) liver genes enriched in oxidative phosphorylation, lipid metabolism and cardiomyopathy pathways; iv) subcutaneous adipose fat IL6 and SELE genes involved in inflammatory and immune system pathways; v) mesenteric adipose fat genes enriched in prolactine signaling, T2DM and PI3K-Akt signaling pathways. (TIFF) [file pone.0279335.s014.tiff]

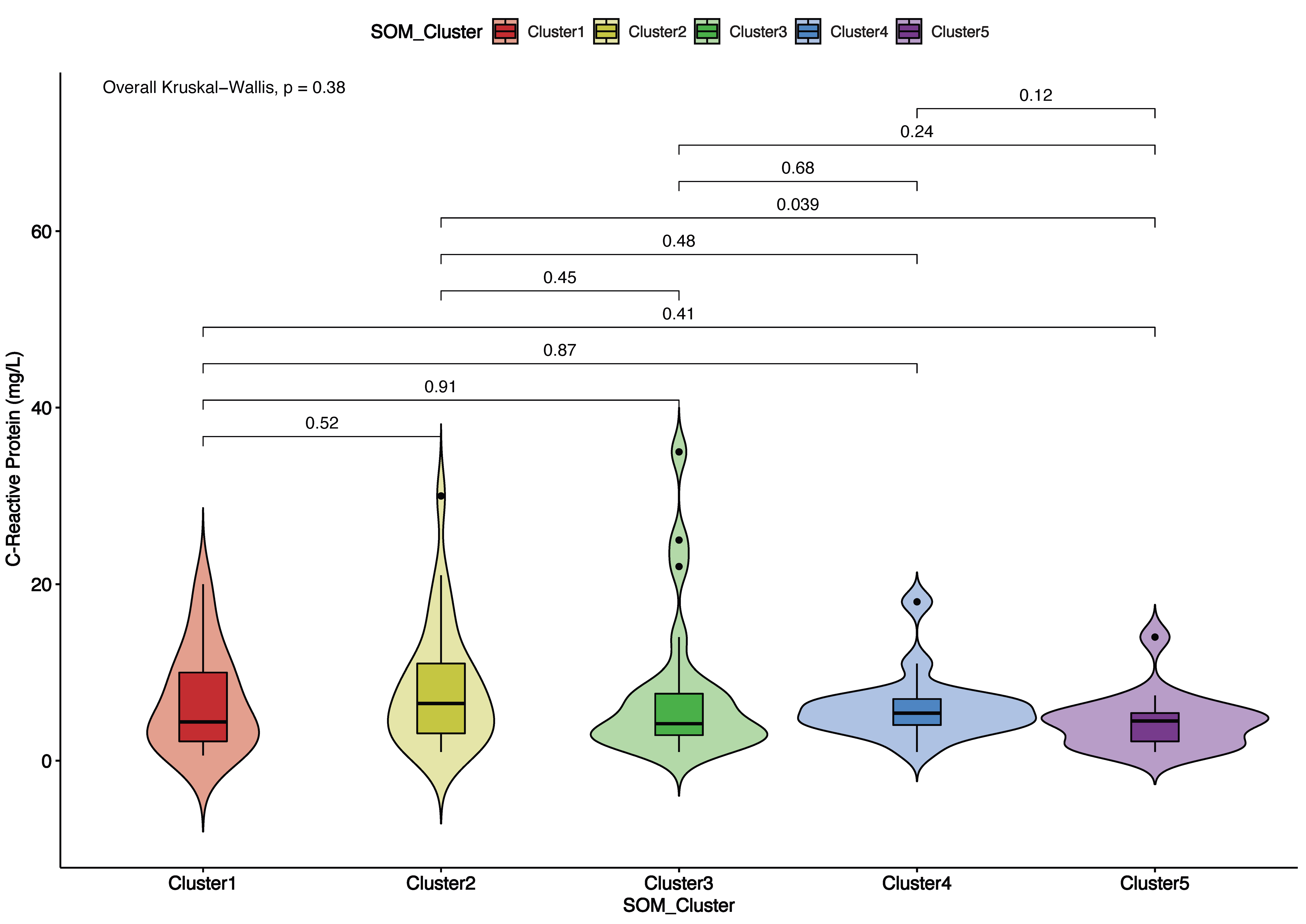

Supplement: S15 Fig — Statistical significance among metabotypes is calculated with Kruskal-Wallis test and p value has been adjusted with FDR. (TIFF) [file pone.0279335.s015.tiff]
